# Swallowing of Pins

**Published:** 1846-07

**Authors:** 


*Swallowing of Pins.*—A maid servant, while engaged in hanging up some curtains, accidentally swallowed a pin. Dr. Neumann, who was immediately sent for, found her pale from fright, and in a state of general tremor. She did not complain of pain in any part, and as she swallowed solids and liquids withont difficulty, Dr. N. persuaded her that she had been probably deceived by her sensation. Her cheerfulness soon returned, and she enjoyed her usual health.

About a year afterwards, Dr. Neumann was suddenly sent for to remove the pin. On his arrival, he ascertained that the female had for some days felt an itching pain in her left side, which she attributed to the presence of the pin. A small pustule was observed over the region of the descending colon, and in this was the pin with the point projecting outwards. It was easily removed by the forceps, and was found to be perfectly clean. It had thus been in the alimentary canal for a year without giving rise to any unfavorable symptom.—*Chem. Gaz., from Casper's Wochenshrift.*

